# The effect of the electric trapping field on state-selective loading of molecules into rf ion traps

**DOI:** 10.1038/s41598-020-74759-6

**Published:** 2020-10-28

**Authors:** Laura Blackburn, Matthias Keller

**Affiliations:** Department of Physics and Astronomy, University of Sussex, Falmer, BN1 9QH UK

**Keywords:** Atomic and molecular interactions with photons, Electronic structure of atoms and molecules

## Abstract

Trapped molecular ions in pure rovibronic states are desirable in experiments ranging from cold chemistry to searches for physics beyond the Standard Model. Resonance-enhanced multiphoton ionisation (REMPI) can be used to prepare molecular ions in specific internal states with high fidelities. However, in the presence of electric fields, ionisation spectra exhibit frequency shifts and the ionisation thresholds are broadened. For this reason, REMPI studies are normally conducted in low and highly homogeneous electric fields, whereas the operating principle of rf ion traps requires electric fields that vary in space and time. In order to investigate the impact of this on the state-selectivity of REMPI in ion traps, we have simulated the expected broadening of the ionisation threshold under various operating conditions of a typical linear Paul trap. In many cases, the width of the ionisation threshold exceeds the separation between rotational energy levels, preventing state-selective ionisation. Careful choice of the trapping and laser parameters during loading can reduce this broadening, enabling state-selective ionisation in some instances. Where this strategy is not sufficient, the broadening can be reduced further by rapidly switching the trapping voltages off and on again during loading. This has been demonstrated experimentally for a Coulomb crystal of $^{40}\hbox {Ca}^+$ ions without descrystallising it.

## Introduction

Trapped molecular ions have developed into indispensable tools with applications spanning cold quantum chemistry^1,2^, quantum computing^3^, metrology^4^ and the search for new physics beyond the Standard Model^5^. In recent years, ion-neutral reactions have been studied with rovibrational quantum-state control^6–9^. In addition, the realisation of novel cooling techniques^10^ and quantum logic detection schemes^11^ has made high-resolution measurements with molecular ions practicable. High-resolution spectroscopy of molecular ions can be used to search for time variation of the proton-to-electron mass ratio^12–18^, the electron’s electric dipole moment^19,20^, parity violation in chiral polyatomic molecules^21,22^ and nuclear-spin-dependent parity violation^23^.

For experiments requiring quantum-state control, molecular ions must be prepared in specific rovibronic states with high fidelity. There are three categories of state preparation method: internal-state pumping, collisional cooling and state-selective ionisation. Internal-state pumping can be employed to significantly increase the population of polar ions in the rovibronic ground state^24–28^. However, this is only possible for a small number of specific molecules with suitable transitions. Alternatively, molecular ions can be prepared in a low-temperature thermal distribution of their internal states through collisional thermalisation with a neutral laser-cooled species^29,30^. While this cooling method can, in principle, be applied to a wide range of molecular ions, charge exchange processes limit the possible combinations of neutral coolant and molecular ion. Finally, photoionisation just above a rovibrational ionisation threshold can produce molecular ions in a specific rovibronic state with purities greater than 99%^31^. Resonance-enhanced multiphoton ionisation (REMPI) has been used to load molecular ions into rf ion traps state-selectively for some species^32–34^. As pure rotational transitions are electric-dipole-forbidden, the state purity of non-polar ions is not lost through interaction with the background blackbody radiation (BBR). While thermalisation with BBR in room-temperature traps limits this approach for polar molecular ions, they can be loaded state-selectively into cryogenic traps where thermal excitation of rotational levels is greatly reduced.

Although photoionisation may be used to load ions into rf traps, the trapping fields can cause a field-induced broadening and lowering of the ionisation threshold^34–36^ which may compromise the state-selectivity of the process. In order to assess the influence of electric trapping fields on the state purity of molecular ions generated via REMPI, we investigated the expected broadening of the ionisation threshold. We found that, while it is possible to load state-selectively for some trapping parameters, field-induced shifts of the ionisation threshold must be mitigated to ensure state-selectivity in many typical trapping fields. We conclude with suggestions for how state-selectivity might be achieved in these circumstances. As a proof of concept, we demonstrate that the trap drive can be rapidly switched off and on again without decrystallising trapped ions.

## Ionisation spectra in electric fields

This study focuses on a two-colour REMPI process. In the first (excitation) step of the scheme, a neutral molecule is resonantly excited to an intermediate state. In the subsequent ionisation step, an electron is removed. Each step can involve the absorption of one or more photons of the same or different energies. Here, we focus on the effect of electric field inhomogeneities on the ionisation step and therefore disregard the details of the REMPI scheme.

In the absence of an external electric field, the ionisation process can be resonantly enhanced by populating autoionising Rydberg states near the ionisation threshold. This leads to clear resonances at which the molecule is predominantly ionised into a specific internal state. These resonances can be spectroscopically well-separated^37^. In this case, molecular ions can be generated in almost any internal state as long as the spectral overlap between the ionisation resonances is small.

In the presence of a homogeneous external electric field, the differential Stark shift of the underlying Rydberg states broadens the ionisation resonances and lowers the ionisation threshold. This leads to a change from distinct resonances to a continuous ionisation spectrum with threshold behaviour (e.g. Fig. 2 in^31^). If the laser for the ionisation step is tuned to or slightly above the ionisation threshold for the desired state, ionic states above this energy are energetically forbidden. Molecular ions will only be generated in states below this threshold. As this is a threshold ionisation process, state-selectivity can only be achieved for the lowest-lying ionic states.

Exciting a specific intermediate state can facilitate preferential ionisation into specific states within the molecular ion. However, in the absence of strict rotational selection rules for the ionisation step of the process, this measure alone is usually not sufficient to achieve high state-selectivity. Certain levels in the ionised molecule can be precluded by careful choice of the initial and intermediate states. For example, the number of accessible states in the ion can be restricted by the symmetry of the wavefunction of the initial state of the neutral molecule. Ionisation of homonuclear molecules between $\Sigma ^{+}_\text {g}$ electronic states in the neutral molecule and ion results in an even-odd staggering of the rotational states due to constraints imposed by the symmetry of the nuclear wavefunction^31^.

The threshold must be well defined to enable state-selective ionisation, requiring a low electric field inhomogeneity and that other broadening mechanisms and the laser line widths are small compared to the rotational constant. Otherwise, molecules at different positions will have different thresholds, producing molecular ions in various internal states. While the conditions on the broadening mechanisms and laser line widths are usually well fulfilled for experiments with nanosecond laser pulses, the electric field inhomogeneity is challenging in rf ion traps. Although highly homogeneous electric fields are possible in spectroscopic set-ups, the principle of rf ion traps is fundamentally based on the inhomogeneity of the trapping field. This can severely compromise the state-selectivity of the ionisation process.

**Figure 1:**
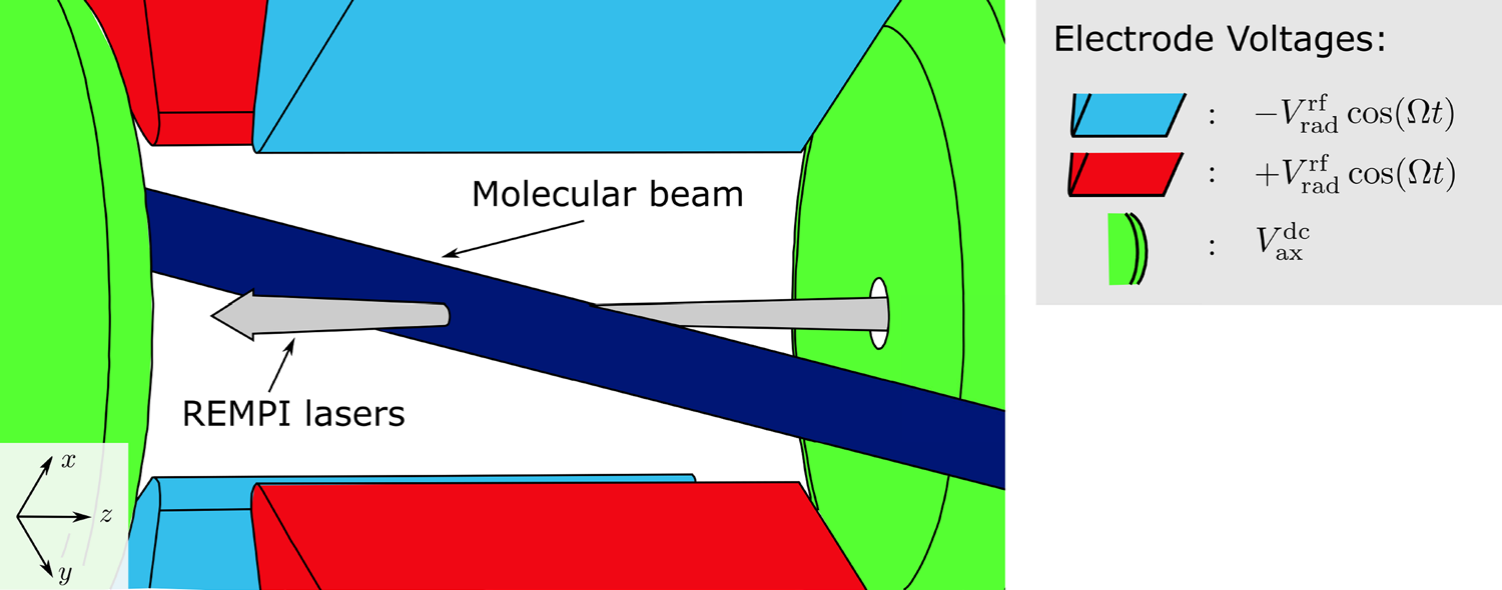
Schematic diagram of a linear rf ion trap. The ionisation lasers are collinear with the trap axis and the molecular beam is aligned perpendicular to it through the centre of the trap. The voltages applied to the electrodes are labelled, consistent with Eq. (1).

## Simulation methods

In a typical linear Paul trap, a rf electric field is used to confine the ions radially and a dc electric field confines the ions along the trap axis. The electric field generated by the trapping electrodes can be expressed in terms of the radial rf voltage, $V_\text {rad}^\text {rf}$, and the axial dc voltage, $V_\text {ax}^\text {dc}$. In Cartesian coordinates, where *x* and *y* form the radial plane and *z* is collinear with the trap axis (see Fig. 1), the magnitude of the total electric field, *E*, is given by^38^

1 $$\begin{aligned} E(x,y,z,t) = \frac{1}{r_0^2} \sqrt{\left( -V_\text {rad}^\text {rf}\cdot \cos (\Omega t)-\eta V_\text {ax}^\text {dc}\right) ^2x^2+\left( V_\text {rad}^\text {rf}\cdot \cos (\Omega t) - \eta V_\text {ax}^\text {dc}\right) ^2y^2 + 4 \left( \eta V_\text {ax}^\text {dc}\right) ^2z^2}, \end{aligned}$$

where *t* is the time, $\Omega$ is the trap drive frequency, $\eta$ is the axial trap efficiency, which accounts for the axial electrode separation and geometry, and $2r_0$ is the effective distance between the radial electrodes, which also takes the deviation from the ideal electrode geometry into account. The origin of the coordinate system is at the centre of the trap.

In a local electric field such as this, the ionisation threshold of neutral molecules is shifted by

2 $$\begin{aligned} \Delta = \alpha \cdot \sqrt{E}, \end{aligned}$$

where $\Delta$ is the wavenumber shift of the ionisation threshold from the zero-field ionisation energy and $\alpha$ is a constant of proportionality that depends on the diabaticity of the ionisation process^36,39,40^. In this paper, we assume that ionisation takes place in the adiabatic regime for the reasons described by Stollenwerk *et al.*^34^ Under this assumption, we take $\alpha$ = 6.1 $\frac{1}{{{\text{cm}}}} \cdot \sqrt {\frac{{{\text{cm}}}}{{\text{V}}}}$^36^.

It can be seen from Eqs. (1) and (2) that the shift of the ionisation threshold strongly depends on the position of the target molecule in the trap. As the molecules and the laser beams have spatial distributions, there will be a distribution of ionisation thresholds. The weighting of a particular ionisation threshold in the distribution is proportional to the probability of ionisation at the corresponding electric field. This, in turn, depends on the spatial and temporal profiles of the ionisation lasers and the density of molecules across the trapping region. In addition to the broadening of the ionisation threshold due to the electric field inhomogeneity, there is an intrinsic broadening of the ionisation resonance due to the differential Stark shift of the Rydberg states. This effect, in addition to deviation of the threshold shift from Eq. (2) for large electric fields, will be neglected in the following. Although these effects can be significant, here we focus only on the effect of ionisation threshold shifts on the ionisation process.

### Simulation scenarios

The following simulations explore the effects of the electric trapping potential and ionisation laser timings on the broadening of the ionisation threshold for a $N+M'$ REMPI process. In the following simulations, we use the low intensity equation^41^

3 $$\begin{aligned} P_\text {ion}^\text {thr} = \chi \rho _\text {mol}I_\text {N}^{N} I_\text {M}^{M}, \end{aligned}$$

where the ionisation probability, $P_\text {ion}^\text {thr}$, is proportional to the molecular density $\rho _\text {mol}$ and the intensities $I_\text {N/M}$ of the two lasers to the power of the numbers of photons, *N* and *M*, absorbed in the excitation and ionisation steps respectively. $\chi$ contains all other details of the ionisation process such as the laser detunings, transition moments and laser polarisations. Because we focus on the structure of the ionisation probability rather than its magnitude, we disregard proportionality constants in the following. We do not consider any other broadening mechanisms. We have considered two scenarios:

1. The neutral molecules are supplied via a molecular beam, perpendicular to the ionisation lasers.
2. The neutral molecules are ionised from a homogeneous background gas.

In both cases, the REMPI lasers are collinear with each other and the trap axis (Fig. 1). In the following, we have assumed that the lasers are Gaussian beams with width $w_\text {N/M}$ and a Gaussian temporal shape with pulse width $\tau _\text {N/M}$, such that

4 $$\begin{aligned} I_\text {N/M}(x, y, z, t) \propto \frac{1}{1 + \big (\frac{2z}{{z_\text {RN/M}}}\big )^2} \cdot \exp \left( -\frac{x^2+y^2}{w_\text {N/M}^2}\right) \cdot \exp \left( -\frac{t^2}{\tau _\text {N/M}^2}\right) , \end{aligned}$$

where ${z_\text {RN/M}}$ is the Rayleigh length of the laser beam. In scenario (1) we use a molecular beam with Gaussian cross section $\rho _\text {mol}(z) \propto \exp (- z^2 / w_\text {beam}^2)$ of width $w_\text {beam}$. Assuming that $w_\text {N/M}<<w_\text {beam}$ and $z_\text {RN/M}>>w_\text {beam}$, Eq. (3) can be approximated by

5 $$\begin{aligned} P_\text {ion}^\text {thr}(x,y,z,t) \propto \exp \left[ -((x - x_\text {offset})^2+(y - y_\text {offset})^2)\left( \frac{{N}}{w_\text {N}^2} +{\frac{{M}}{w_\text {M}^2}}\right) -\frac{(z - z_\text {offset})^2}{w_\text {beam}^2} -(t - t_\text {offset})^2\left( \frac{{N}}{\tau _\text {N}^2} +{\frac{{M}}{\tau _\text {M}^2}}\right) \right] , \end{aligned}$$

The REMPI lasers can be displaced from the centre of the trap in the *x* and *y* directions by distances $x_\text {offset}$ and $y_\text {offset}$ respectively and the molecular beam can be displaced in the *z* direction by $z_\text {offset}$. It is assumed that these displacements are significantly smaller than the dimensions of the trap. The relative timing of the laser pulses to the phase of the rf field can also be varied by changing the delay time, $t_\text {offset}$, between the peak of an rf oscillation and the arrival time of the REMPI lasers. In scenario (2), the molecular density is constant and Eq. (3) becomes

6 $$\begin{aligned} \begin{aligned} P_\text {ion}^\text {thr}(x,y,z,t)&\propto \Bigg (\frac{1}{1 + \big (\frac{2(z - z_\text {offset})}{z_\text {RN}} \big )^2} \Bigg )^{N} \cdot \Bigg (\frac{1}{1 + \big (\frac{2(z - z_\text {offset})}{z_\text {RM}} \big )^2} \Bigg )^{M} \\&\quad \cdot \exp \left[ -((x - x_\text {offset})^2+(y - y_\text {offset})^2)\left( \frac{{N}}{w_\text {N}^2}+ \frac{{M}}{w_\text {M}^2}\right) -(t -t_\text {offset})^2\left( \frac{N}{\tau _\text {N}^2}+ \frac{M}{\tau _\text {M}^2}\right) \right] . \end{aligned} \end{aligned}$$

where $z_\text {offset}$ is the *z*-displacement of the laser focuses from the centre of the trap.

The probability of ionising at a given field magnitude *E* is given by the integral of the ionisation probability over the surface, $S_\text {E}$, defined by the ellipsoid of constant electric field described in Eq. (1), where

7 $$\begin{aligned} P_\text {ion}(E) \propto \int \oint P_\text {ion}^\text {thr}(x,y,z,t) \text {d}S_\text {E} \text {d}t. \end{aligned}$$

In general, this integral needs to be solved numerically, using the ellipsoidal parameterisation of the electric field

8 $$\begin{aligned} {\vec {\mathbf{r }}}(u, v) = a \cos (u) \sin (v) \cdot {\hat{\mathbf{i }}} + b \sin (u) \sin (v) \cdot {\hat{\mathbf{j }}} + c \cos (v) \cdot {\hat{\mathbf{k }}}, \end{aligned}$$

where *u* is the azimuth angle from the positive *x*-axis and *v* is the polar angle with respect to the *z*-axis to positions on the surface of the ellipsoid; $\hat{\mathbf{i }}$, $\hat{\mathbf{j }}$ and $\hat{\mathbf{k }}$ are unit vectors along the $+x$, $+y$ and $+z$ directions respectively; and *a*, *b* and *c* are parameters that depend on the electric field. In the general case, where all electrode voltages are considered,

9 $$\begin{aligned} a = \frac{E \cdot r_0^2}{\left( \eta V_\text {ax}^\text {dc}+ V_\text {rad}^\text {rf}\cdot \cos (\Omega t)\right) },\; b = \frac{E \cdot r_0^2}{\left( \eta V_\text {ax}^\text {dc}-V_\text {rad}^\text {rf}\cdot \cos (\Omega t) \right) },\; c = \frac{E \cdot r_0^2}{\left( 2 \eta V_\text {ax}^\text {dc}\right) }. \end{aligned}$$

With these substitutions, the integral becomes

10 $$\begin{aligned} P_\text {ion}(E) = \int _{-\infty }^{\infty } \int _0^{\pi } \int _0^{2 \pi } P_\text {ion}^\text {thr}(x(u, v), y(u, v), z(u, v), t) {\left\| \frac{\partial {\vec {\mathbf{r }}}}{\partial u} \times \frac{\partial {\vec {\mathbf{r }}}}{\partial v}\right\| } \text {d}u \text {d}v \text {d}t, \end{aligned}$$

where the term ${\left\| ...\right\| }$ represents the Jacobi determinant. From Eq. (2), the electric field can be replaced by

11 $$\begin{aligned} E = \frac{\Delta ^2}{\alpha ^2}, \end{aligned}$$

to convert from electric field strength to the shift of the ionisation threshold from the zero-field value. In order to make the results of our investigations independent of the exact electrode geometry, and hence applicable to the most common linear rf ion traps, we replace the applied voltages with the trap *q*-parameter, *q*, and the axial secular frequency, $\omega _z$:

12 $$\begin{aligned} V_\text {rad}^\text {rf} = \frac{m q r_0^2 \Omega ^2}{2 Q},\quad V_\text {ax}^\text {dc} \eta = \frac{m \omega _z^2 r_0^2}{2 Q}, \end{aligned}$$

where *Q* is the charge of the ion produced in the ionisation process and *m* is its mass^38^. In this way, Eq. (10) only depends on *q*, $\Omega$ and $\omega _z$ and the results of the numerical analysis can be easily scaled to other ion traps.

It is informative to consider the effects of certain special cases. In addition to the general case (described by Eqs. (8)–(10)), we have considered the effects of varying the radial confinement, the timing of the REMPI laser pulses and the dc voltage applied to the axial electrodes. As these special cases consider the rf and dc voltages separately, it is possible to simplify the parameterisation of the electric field in Eqs. (8) and (9). The subsections below describe the simplified parameterisations used in the simulations.

#### Radial electric field

The radially applied electric field increases linearly from the trap centre and oscillates in time. If the laser pulses are timed to coincide with a specific phase of the rf drive and the ionisation lasers pass through the centre of the trap ($x_\text {offset} = y_\text {offset} = 0$), the integration over the spatial degrees of freedom has an analytic solution, but the integration with respect to time must be done numerically. Equation (10) becomes

13 $$\begin{aligned} P_\text {ion}(\Delta ) \propto \Delta ^2 {\int \limits _{ t_\text{{offset}} - \infty }^{ t_\text{{offset}} + \infty }} \frac{1}{|\cos (\Omega t)|}\exp \Bigg [ - \Bigg (\frac{\Delta ^2}{\alpha ^2} \cdot \frac{r_0^2}{V^\text {rf}_\text {rad} \cos (\Omega t)} \Bigg )^2 \cdot \Bigg (\frac{N}{w_\text {N}^2} + \frac{M}{w_\text {M}^2}\Bigg ) - (t - t_\text{{offset}})^2 \Bigg (\frac{N}{\tau _\text {N}^2} + \frac{M}{\tau _\text {M}^2}\Bigg ) \Bigg ] \text {d}t. \end{aligned}$$

However, if the lasers are offset from the centre of the trap, Eq. (10) must be solved numerically. Due to the radial symmetry of the electric field produced by the rf electrodes, it is possible to simplify this by using the circular parameterisation

14 $$\begin{aligned} {\vec {\mathbf{r }}}(u) = a_\text{{rad}} \cos (u) \cdot {\hat{\mathbf{i }}} + a_{\text{rad}} \sin (u) \cdot {\hat{\mathbf{j }}}, \end{aligned}$$

where

15 $$\begin{aligned} a_\text{{rad}} = \frac{E_\text {rad} r_0^2}{V_\text {rad}^{\text {rf}} \cdot |\cos (\Omega t)|}, \end{aligned}$$

and $E_\text {rad}$ is the time-dependent electric field applied by the radial electrodes (see Eq. (1)).

#### Axial electric field

The dc electric field varies along the trap axis as well as radially, but is independent of time. The axially applied electric field, $E_\text {ax}$, from Eq. (1), can be written in terms of the axial secular frequency, $\omega _\text {z}$, to give

16 $$\begin{aligned} E_\text {ax} = \frac{m\omega _\text {z}^2}{2 Q}\sqrt{r^2+4z^2}, \end{aligned}$$

where $r^2 = x^2 + y^2$. The circular symmetry in the radial plane allows the parameterisation

17 $$\begin{aligned} {\vec {\mathbf{r }}}(u, v) = a_{\text{ax}} \cos (u) \sin (v) \cdot {\hat{\mathbf{i }}} + a_{\text{ax}} \sin (u) \sin (v) \cdot {\hat{\mathbf{j }}} + b_{\text{ax}} \cos (v) {\hat{\mathbf{k }}}, \end{aligned}$$

where

18 $$\begin{aligned} a_\text{{ax}} = \frac{2 E_\text {ax} Q}{m \omega _\text {z}^2},\;\; b_\text{{ax}} = \frac{E_\text {ax} Q}{m \omega _\text {z}^2}. \end{aligned}$$

## Results

The following subsections present the effect of the trap parameters and laser pulse timings on the broadening of the ionisation threshold. This broadening is quantified by the full-width at half-maximum (FWHM) of the ionisation threshold. These simulations explore the effects of the radial confinement, laser pulse timings and axial confinement separately before considering the general case, where voltages are applied to both the radial and axial electrodes. The state-selectivity of a chosen REMPI process can be assessed by comparing the width of the ionisation threshold to the splitting of energy levels within the molecular ion. As an example ionisation process, we take the case of 2+1’ REMPI of $^{14}$N$_2$ molecules in a linear Paul trap.

Finally, we present the rapid switching off of the trap drive, without decrystallising a Coulomb crystal of Ca$^+$ ions, to demonstrate the potential of the method to mitigate the broadening observed due to the trapping field.

**Figure 2:**
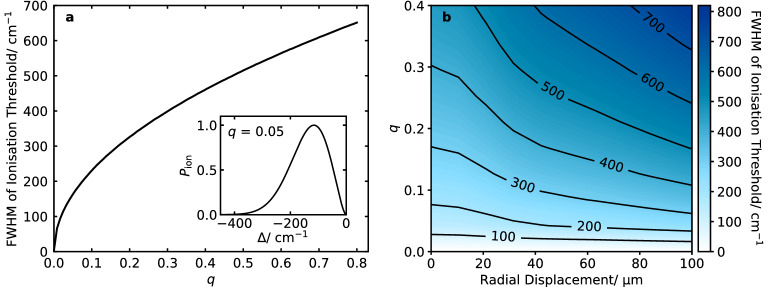
Ionisation threshold distributions were simulated for 2+1’ REMPI of $^{14}\hbox {N}_2$ with a trap drive frequency $\Omega = 2\pi \cdot 20$ MHz and ionisation laser beamwaists $w_\text {N/M} = 50\,\upmu \hbox {m}$. The laser pulses were timed to the zero-crossing of the rf field, with temporal widths $\tau _\text {N/M} =$ 5 ns. (**a**) Broadening of the ionisation threshold for different *q* parameters, where the REMPI lasers pass through the centre of the trap. Inset, the normalised ionisation threshold distribution for $q=0.05$. (**b**) Broadening of the ionisation threshold for different *q* values and radial displacements of the REMPI lasers from the trap centre.

### Effect of the radial confinement

The width of the threshold distribution shows a square-root behaviour for increasing *q*-parameter (Fig. 2a). With a trap drive frequency of $2\pi \cdot$ 20 MHz, the broadening exceeds $100\hbox { cm}^{-1}$ for *q* values as low as 0.05. This is significantly larger than the typical rotational constants of molecules.

The Gaussian laser profiles and size of the surface element result in a shell-like ionisation threshold distribution (Fig. 2a) with no photoionisation at the centre of the trap ($\Delta = 0\hbox { cm}^{-1}$) due to the small volume element. From the centre of the trap, the photoionisation probability increases due to the increased ionisation volume and then decreases again due to the lasers’ intensity profiles. While the FWHM reflects the broadening of the threshold distribution, it neglects the shift of the threshold which can be reasonably large.

For a radial displacement of the photoionisation lasers, the broadening of the ionisation threshold increases with the displacement from the trap centre (Fig. 2b). For a displacement of $100\, \upmu \hbox {m}$ from the trap centre, the increase in the broadening is already significant for higher confinements. The ionisation threshold broadening is therefore sensitive to small misalignments of the ionisation lasers in the trap. Depending on the design of the trap, sufficient alignment precision may be difficult within the tolerance of the ionisation scheme.

**Figure 3:**
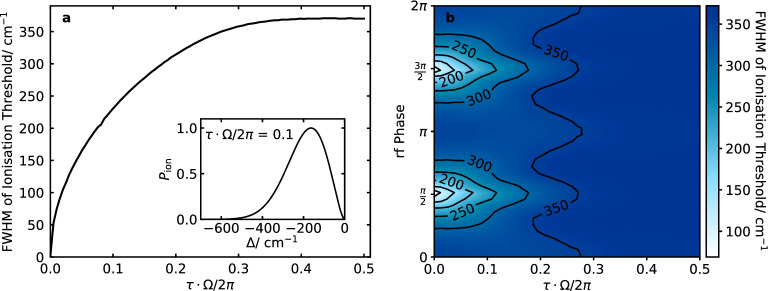
Ionisation threshold distributions were simulated for 2+1’ REMPI of $^{14}\hbox {N}_2$ for $q = 0.1$ and no radial displacement of the REMPI lasers. (**a**) Broadening of the ionisation threshold for different laser pulse durations where the lasers are timed to coincide with the zero-crossing of the rf field. Inset, the normalised ionisation threshold distribution for a laser pulse duration of 10% of the trap drive period. (**b**) Broadening of the ionisation threshold for different laser pulse durations and phases of the rf trap drive relative to the arrival times of the ionisation lasers.

### Effect of the laser pulse timing

With the laser pulses timed to coincide with the zero-crossing of the rf drive, the FWHM of the ionisation threshold increases with the temporal pulse width (Fig. 3a). For a pulse width of $\sim 1\%$ of the period of the rf field, the broadening of the ionisation threshold is approximately $50\hbox { cm}^{-1}$ for a trap *q* value of 0.1. As the temporal pulse width increases, the rate of broadening decreases until it begins to plateau at around 50% of the trap drive period, as the laser pulses begin to extend significantly beyond one period of the rf drive. When the rf phase at which the laser pulses arrive is varied, the periodicity of the broadening is notable for pulse durations less than 20% of the trap drive period (Fig. 3b). The plateauing of the ionisation threshold widths can be seen for all phases. For larger pulse durations the effect of the trap drive phase is minimal.

**Figure 4:**
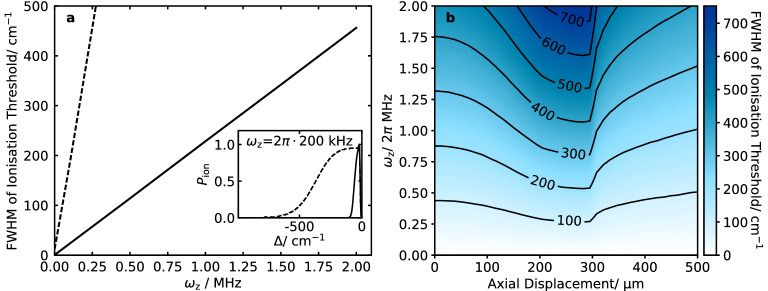
Ionisation threshold distributions were simulated for 2+1’ REMPI of $^{14}\hbox {N}_2$. (**a**) Broadening of the ionisation threshold for different axial secular frequencies for loading from a molecular beam with waist $w_\text {beam} = 250\,\upmu \hbox {m}$ (solid line) and from a homogeneous background gas (dashed line). In both scenarios, $z_\text {offset} = 0$. Inset, the normalised ionisation threshold distributions for an axial secular frequency of $2\pi \cdot 200$ kHz. (**b**) Broadening of the ionisation threshold for different axial secular frequencies and axial displacements from the trap centre for loading from a molecular beam with waist $w_\text {beam} = 250\,\upmu \hbox {m}$.

### Effect of the axial confinement

#### Scenario 1: molecular beam

The broadening of the ionisation threshold increases linearly with the axial secular frequency (Fig. 4a). Here, by way of example, we considered ionisation from a molecular beam of waist $w_\text {beam} = 250 \,\upmu \hbox {m}$ passing through the centre of the trap. For these parameters, the increase in the broadening with axial confinement is approximately $225\hbox { cm}^{-1}$ $\hbox {MHz}^{-1}$ which is significant compared to the splitting of rotational levels in a typical molecular ion. The shape of the ionisation threshold differs from that seen in the radial case due to the different electric field gradients along the trap axis and out radially from the trap centre. The result is an initial spike followed by a slow decrease (Fig. 4a). Increasing the displacement of the molecular beam from the centre of the trap leads to an initial increase in the broadening followed by a decrease as the displacement exceeds the width of the beam (Fig. 4b). This is a consequence of the square-root relationship between the electric field and the corresponding spectral shift (Eq. (2)). At higher electric fields, the gradient of the shift is smaller so the range of shifts seen by the target molecules is smaller. While smaller peak widths can be obtained for large offsets from the trap centre, it is not desirable to ionise there due to the large energy acquired by ions produced in strong electric fields. It is therefore preferable to ionise as close as possible to the centre of the trap.

#### Scenario 2: homogeneous background gas

In scenario (2), we consider ionisation laser beam waists $w_\text {N/M} = 50\,\upmu \hbox {m}$ and wavelengths $\lambda _\text {N} = 255\hbox { nm}$, $\lambda _\text {M} = 212\hbox { nm}$.

The FWHM of the ionisation threshold increases linearly with the axial secular frequency, but more rapidly than in scenario 1.). The gradient in this case is $\sim 1,800\hbox { cm}^{-1}$ $\hbox {MHz}^{-1}$. This is because the intense part of the laser beam extends much further than the width of the molecular beam previously considered. We do not consider the impact of displacing the focus from the trap centre, but the effect is likely to be smaller due to the larger extent of the ionisation volume in the axial direction.

**Figure 5:**
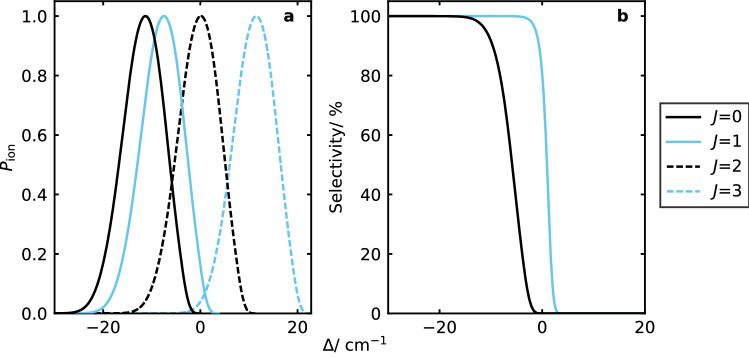
(**a**) Ionisation threshold distributions shown for the first four rotational energy levels ($J=0, 1, 2, 3$) in the $^{14}\hbox {N}_2^+$ ion where zero shift corresponds to the zero-field ionisation threshold for the lowest rotational level. The trap parameters were *q* = 0.01, $\omega _\text {z} =2 \pi \cdot 50$ kHz and the trap drive frequency was $\Omega = 2 \pi \cdot 5$ MHz. The laser pulse durations were $\tau _\text {M/N} = 5\hbox { ns}$ and the ions were loaded from a molecular beam of width $250\, \upmu \hbox {m}$. (**b**) State-selectivity of ionisation into the $J=0$ and $J=1$ levels for different detunings of the ionisation laser. For each, we consider only the contribution from the next possible rotational state.

**Figure 6:**
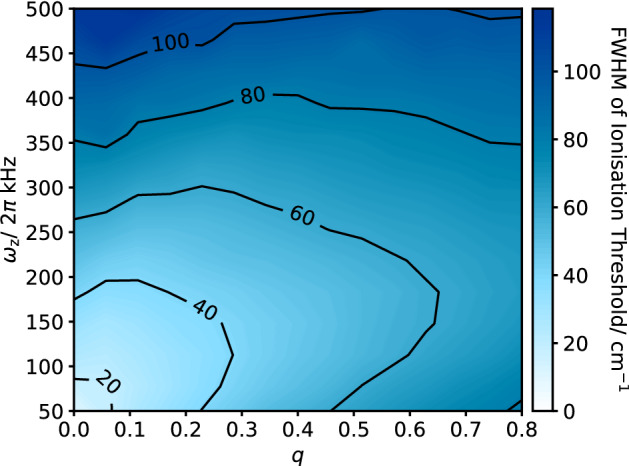
Broadening of the ionisation threshold for different axial and radial confinements. Ionisation threshold distributions were simulated for loading from a molecular beam with beam waist $w_\text {beam} = 250\,\upmu \hbox {m}$ into a trap with drive frequency $\Omega = 2 \pi \cdot 5$ MHz. The laser pulses were timed to the zero-crossing of the rf field, with temporal pulse widths $\tau _{N/M}=5$ ns.

### Combined effect of the axial and radial confinement

For the ionisation process considered, ionisation is only possible into rotational states that are either only odd or only even, due to the symmetry of the nuclear wavefunction. The rotational constant for $^{14}\hbox {N}_2^+$ is $1.9\hbox { cm}^{-1}\;$^42^. For trap parameters of $\Omega = 2 \pi \cdot 5$ MHz, $q = 0.01$ and $\omega _\text {z} = 2 \pi \cdot 50$ kHz, the ionisation threshold distributions are sufficiently well-separated to allow rotationally selective ionisation into either the *J*=0 or *J*=1 state (Fig. 5a,b), depending on the initial state of the molecule. Higher rotational levels have not been included for the sake of simplicity, but would reduce the maximum state purity that could be achieved in cases where there is spectral overlap. Although a high degree of state-selectivity can be achieved (Fig. 5b), this must be balanced against the probability of ionisation (Fig. 5a) at the laser frequencies where this is possible.

While the broadening increases linearly for the axial confinement and with a square-root behaviour for the radial case, the combined broadening in the presence of both the axial and radial voltages is more complicated (Fig. 6) because the radial component of the dc confinement partially compensates the rf electric field.

**Figure 7:**
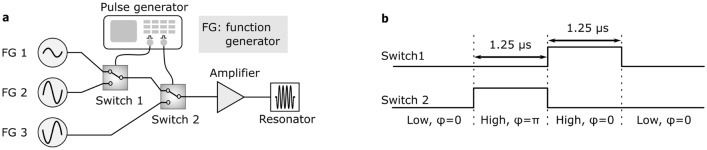
(**a**) The set-up used to rapidly switch the rf trapping field off and on again. Initially, the resonator circuit is driven by a low amplitude signal from FG 1. The drive source is switched to FG 3 which supplies a high-amplitude signal, with a phase difference of $\pi$, which interferes with the intra-resonator field. On reaching the minimum amplitude, the resonator input is switched to FG 2, a high-amplitude signal in-phase with the original drive, which restores the trapping field. Finally, the drive from FG 1 is reinstated. (**b**) The pulse sequence used to switch between FG 1, FG 2 and FG 3.

### Switching of the trap confinement

The previous subsections demonstrate the importance of minimising the electric field when photoionising molecules in rf traps. Whilst in some cases it may be possible to work with trapping fields low enough to achieve the desired state-selectivity, an alternative is to lower the trapping field only for the duration of the loading process. If the trap voltages are lowered and raised slowly, there is a risk of losing ions that were already trapped, due to small disturbances such as collisions with residual background gas. Switching the trap off for a very short time reduces this risk, but is technically more complex. The dc axial confinement can be switched off reasonably easily, but this is not necessarily the case for the rf confinement. One way to facilitate fast trap switching is to use a digital trap in which the rf voltage switches between three discrete levels to resemble an oscillation (see e.g^43^). Within the rf cycle, there can be a period when the voltage on the rf electrodes is zero and applying the REMPI lasers during this period can be used for state-selective loading. Alternatively, for an ion trap driven via a resonant circuit, the resonant circuit can be dumped by a built-in high voltage switch. After the resonator field is extinguished, no voltage is present at the rf electrodes. However, in this case, the build-up of the voltage on the rf electrodes is limited by the Q factor of the resonator which can make the trap unstable during the recovery of the trapping potential. Both methods are well suited for low frequency ion traps but are difficult to implement for high frequencies.

**Figure 8:**
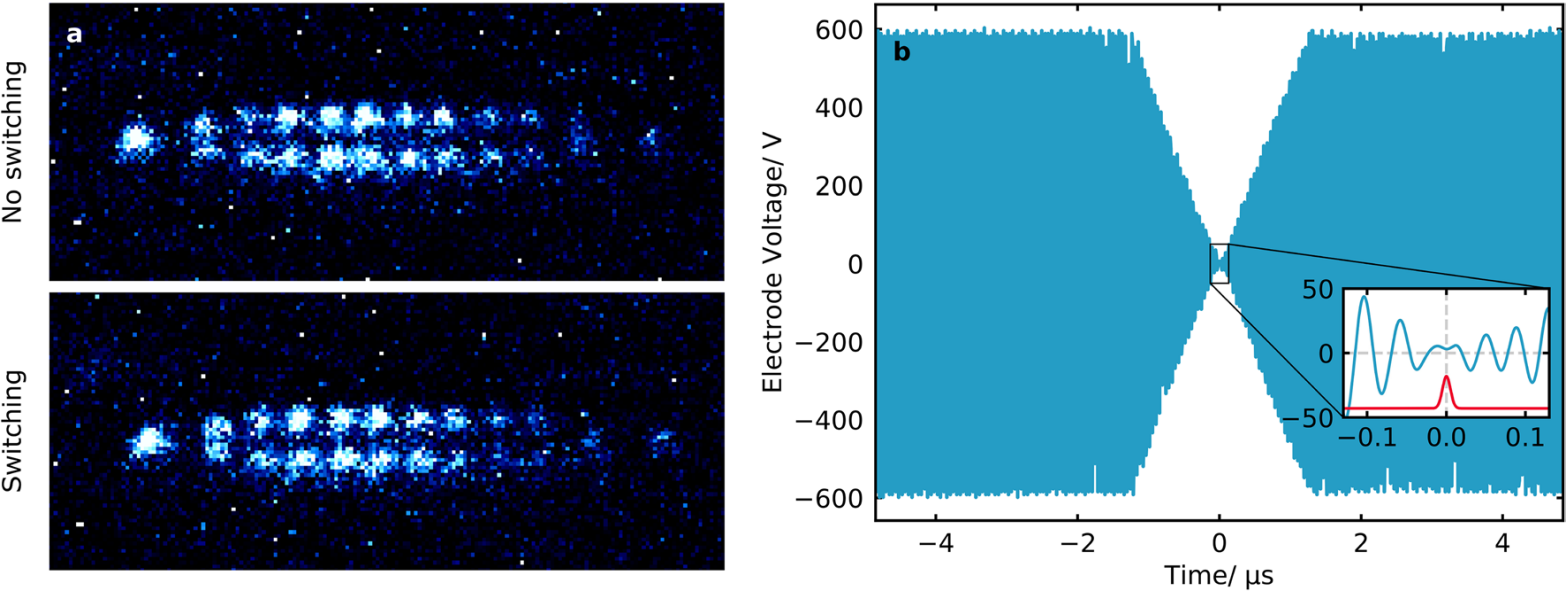
(**a**) False-colour images of 3d Coulomb crystals with ordinary trap operation and whilst the rf amplitude of the trap was rapidly switched at a rate of 2 Hz. (**b**) The voltage on the rf electrodes as the trap was rapidly switched at a rate of 2 Hz for a trap drive frequency of 22 MHz using the set-up in Fig. 7a. The voltage was measured indirectly with a pick-up coil within the resonator. Inset, the electrode voltage around the minimum (blue) and an example laser pulse of arbitrary amplitude with temporal width $\tau$ = 5 ns (red), synchronised to the minimum of the rf voltage. The inset electrode voltage data were treated with a Fourier filter to remove high-frequency noise.

In this work, to achieve more rapid switching, the intra-circuit field was interfered with the driving field (Fig. 7a). By switching the phase of the trap drive by $\pi$ and increasing its amplitude, the resonator field was eliminated much faster than the natural decay rate. Similarly, by applying a strong drive pulse, the rf field was recovered before reducing the drive amplitude back to the stationary value (Fig. 7b). This was performed at a rate of 2 Hz with a 3d Coulomb crystal of $^{40}\hbox {Ca}^+$ ions without de-crystallising it (Fig. 8a). Here, the switch-off time was approximately $1\,\upmu \hbox {s}$ (Fig. 8b). This time was sufficiently short to retain the ions, but faster switching could be achieved by increasing the relative amplitude of the high amplitude drives. The trap switch-off during the dummy laser pulse in Fig. 8b reduces the electric field amplitude during the laser pulse by a factor of 400 which in turn reduces the broadening of the ionisation threshold by a factor of 20. This switching, in conjunction with a careful choice of trap parameters, could facilitate rotationally state-selective REMPI in an ion trap.

## Discussion

In order to achieve rovibronic state-selectivity, the broadening of the ionisation threshold due to the electric trapping field must be smaller than the rotational state splitting of the molecular ion during the loading process. For ion traps with high secular frequencies and a high drive frequency, the electric field inhomogeneity significantly broadens the ionisation threshold, hampering rotational state-selectivity.

Synchronising the laser pulses with the minimum of the rf drive can significantly reduce the broadening. However, this strategy is most effective when the pulse duration is significantly shorter than the period of the rf drive (Fig. 3b), favouring short laser pulses in conjunction with low trapping frequencies. Another way to reduce the broadening is to shrink the spatial region in which ionisation can take place. In the radial plane, this can be done by tightening the focuses of the ionisation lasers. However, in set-ups where dye lasers are used, very tight focusing is challenging due to their poor beam profile. In the axial direction, loading from a molecular beam rather than from a homogeneous background gas has been shown to reduce the broadening for typical laser parameters by a factor of eight for a molecular beam width of $250\,\upmu \hbox {m}$ (Fig. 4a). An additional consideration is that, if loading from a background gas, collisions with the produced ions cannot be prevented. Thermalisation of the rotational state and charge-exchange reactions can cause loss of the produced state. To further reduce the broadening for two-colour and multi-colour REMPI processes, it may be possible to cross the ionisation lasers so that ions are only produced in the volume where they intersect. For the trap we have considered, this would reduce the ionisation region along the axis of the trap, reducing the broadening due to the dc axial electrode voltage. If these steps are inadequate, a final strategy would be to minimise the voltages applied to the electrodes during loading. This can be done by choosing to work at lower confinements during loading. However, where it is not possible to trap with low enough electric fields to resolve the ionisation thresholds for different rovibrational levels, rapid switching of the trap drive may be necessary.

## Conclusions

The distribution of ionisation thresholds across the trapping region in a typical rf trap can be broad enough to prevent state-selective ionisation of molecules. The extent of the broadening and its impact depend on the specific target molecular ion, the trapping parameters and the level of selectivity required. While some ion traps already operate with sufficiently low drive frequencies and electrode voltages, many experiments require high confinements which must be adjusted whilst loading to obtain a high purity of ions in the desired internal state. In these cases, the width of the ionisation threshold can be reduced by adjusting the trap parameters or by rapidly switching the trap off and on again to coincide with the timing of the ionisation lasers. For state-selective REMPI in an ion trap, the choice of trap parameters is crucial and, by employing other mitigation techniques such as trap switching, loading of high frequency traps is feasible.

## Data Availability

The data plotted in Figs. 2–6, 8b can be found at 10.25377/sussex.13060295. The simulation code used in this work can be found at 10.25377/sussex.13063628.
